# Unsupervised Learning and Clustered Connectivity Enhance Reinforcement Learning in Spiking Neural Networks

**DOI:** 10.3389/fncom.2021.543872

**Published:** 2021-03-04

**Authors:** Philipp Weidel, Renato Duarte, Abigail Morrison

**Affiliations:** ^1^Institute of Neuroscience and Medicine (INM-6) & Institute for Advanced Simulation (IAS-6) & JARA-Institute Brain Structure-Function Relationship (JBI-1 / INM-10), Research Centre Jülich, Jülich, Germany; ^2^Department of Computer Science 3 - Software Engineering, RWTH Aachen University, Aachen, Germany

**Keywords:** unsupervised learning, reinforcement learning, spiking neural network, neural plasticity, clustered connectivity

## Abstract

Reinforcement learning is a paradigm that can account for how organisms learn to adapt their behavior in complex environments with sparse rewards. To partition an environment into discrete states, implementations in spiking neuronal networks typically rely on input architectures involving place cells or receptive fields specified *ad hoc* by the researcher. This is problematic as a model for how an organism can learn appropriate behavioral sequences in unknown environments, as it fails to account for the unsupervised and self-organized nature of the required representations. Additionally, this approach presupposes knowledge on the part of the researcher on how the environment should be partitioned and represented and scales poorly with the size or complexity of the environment. To address these issues and gain insights into how the brain generates its own task-relevant mappings, we propose a learning architecture that combines unsupervised learning on the input projections with biologically motivated clustered connectivity within the representation layer. This combination allows input features to be mapped to clusters; thus the network self-organizes to produce clearly distinguishable activity patterns that can serve as the basis for reinforcement learning on the output projections. On the basis of the MNIST and Mountain Car tasks, we show that our proposed model performs better than either a comparable unclustered network or a clustered network with static input projections. We conclude that the combination of unsupervised learning and clustered connectivity provides a generic representational substrate suitable for further computation.

## 1. Introduction

Neural systems learn from past experience, gradually adapting their properties according to processing requirements. Be it in a merely sensory-driven situation or in high-level decision making processes, a key component of learning is to develop adequate and usable internal representations, allowing the system to assess, represent and use the current state of the environment in order to take actions that optimize expected future outcomes (Sutton and Barto, 2018).

While these principles have been extensively exploited in the domain of machine learning, leading to highly proficient information processing systems, the similarities with the biophysical reality are often merely conceptual. The standard approach to learning in artificial neural networks has been end-to-end supervised training with error backpropagation (LeCun et al., 2015; Kriegeskorte and Golan, 2019). However, despite the proficiency of these algorithms, their plausibility under biological constraints is highly questionable (Nikolić, 2017; Marcus, 2018, but see e.g., Marblestone et al., 2016; Lillicrap and Santoro, 2019; Richards et al., 2019 for counterarguments). Whereas a growing body of literature has focused on bridging this divide by adjusting error backpropagation to make it more biophysically compatible (e.g., Sacramento et al., 2018; Bellec et al., 2019; Whittington and Bogacz, 2019), there remains a disconnect between these approaches and real neuronal and synaptic dynamics.

Biological neural networks operate with discrete pulses (spikes) and learn without explicit supervision. Synaptic efficacies are adjusted to task demands relying on local information, i.e., the activity of pre- and post-synaptic neurons, as well as unspecific neuromodulatory gating factors (Porr and Wörgötter, 2007; Frémaux and Gerstner, 2016). As such, any model of the acquisition of internal representations ought to comply with these (minimal) criteria, gradually shaping the system's properties to learn an adequate partitioning of the state space in a manner that allows the system to operate under different task constraints. Furthermore, these learning processes ought to ensure generalizability, allowing the same circuit to be re-used and operate on different input streams, extracting the relevant information from them and acquiring the relevant dynamical organization according to processing demands, in a self-organized manner.

Modeling studies have employed a variety of strategies to allow the system to internally represent the relevant input features (also referred to as environmental states, in the context of reinforcement learning). This can be done, for example, by manually selecting neuronal receptive fields (Potjans et al., 2009, 2011; Jitsev et al., 2012; Frémaux et al., 2013; Friedrich et al., 2014) according to a pre-specified partition of the environment or by spreading the receptive fields uniformly, in order to cover the entire input space (Frémaux et al., 2013; Jordan et al., 2017). These example solutions have major conceptual drawbacks. Manually partitioning the environmental state space is by definition an *ad hoc* solution for each task; whereas uniformly covering the whole input is a more generic solution, it can only be achieved for relatively low-dimensional input spaces. In both cases, the researcher imposes an assumption about the appropriate resolution of partitioning for a given task, and thereby implicitly also affects the learning performance of the neural agent. Both approaches are thus inflexible and restricted in their applicability.

It seems parsimonious to assume that the development of adequate internal states capturing relevant environmental features emerges from the way in which the input is projected onto the circuit. In the reservoir computing paradigm, the input projection acts as a non-linear temporal expansion (Schrauwen et al., 2007; Lukoševičius and Jaeger, 2009). Relatively low-dimensional input streams are thus non-linearly projected onto the circuit's high-dimensional representational space. Through this expansive transformation, the neural substrate can develop suitable dynamic representations (Duarte and Morrison, 2014; Duarte et al., 2018) and resolve non-linearities such that classes that are not linearly separable in the input space can be separated in the system's representational space. This property relies on the characteristics of the neural substrate, acting as a non-linear operator, and the ensuing input-driven dynamics (Maass et al., 2002).

The output of reservoir computing models is commonly a simple (typically linear) supervised readout mechanism, trained on the circuit's dynamics to find the pre-determined input-output mappings. Naturally, such supervised readouts are not intended to constitute realistic models of biological learning (Schrauwen et al., 2007), but instead constitute a metric to evaluate the system's processing capabilities. In a biological system, one would expect the output projections of a reservoir to adapt in response to local information such as pre- and post-synaptic activity, possibly incorporating a global, diffusive neuromodulatory signal. A complication here is that synaptic learning that largely depends on the spiking activity of a pair of neurons is inevitably susceptible to the stochastic nature of that activity.

In this manuscript, we address the above issues of partitioning the input space and learning output projections in a biologically plausible fashion. We introduce a novel class of spiking neural network model, consisting of an input layer, a representation layer based on a balanced random network of spiking neurons, and an output layer. In contrast to classical reservoir computing models (Jaeger, 2001; Maass et al., 2002, 2003), the input projections are subject to unsupervised learning (Tetzlaff et al., 2013), and the output projections are subject to a dopamine-modulated reinforcement learning rule rather than supervised learning. Furthermore, we introduce structure in the recurrent connections within the representation layer, in the form of clustered synaptic connectivity (Rost et al., 2018; Rostami et al., 2020), which is a biologically well-motivated circuit motif (Song et al., 2005; Perin et al., 2011). We demonstrate that this feature, in combination with unsupervised learning of the input projections, substantially boosts the computational performance of the network. The clusters become specialized, in a self-organized fashion, for features of the input space, thus allowing stable representations of the input to emerge that support a linear separation. The low-dimensional dynamics of the clustered network (Litwin-Kumar and Doiron, 2014) provide a stable basis for the three-factor plasticity rule implemented by the output projections to learn appropriate input-output mappings using a reinforcement learning strategy.

We first demonstrate, using the XOR task, the capacity of the unsupervised learning rule to resolve non-linearities in the input, allowing the representation layer to generate linearly separable activity. We then investigate the performance of the full model on a 3-digit MNIST task. We show that unsupervised learning in the input projections allows the output projections to learn the correct classifications with a high degree of accuracy even though the learning is driven by a reinforcement signal (correct/incorrect) rather than the supervisory signal (identification of correct class) more commonly used for classification tasks. The presence of the clusters in the representation layer cause the network to converge more quickly, and to a higher performance, than the corresponding unclustered network. The clustered network with plastic input projections resolves the non-linearities, captures the intra- and inter-class variance and elegantly deals with the highly overlapping inputs of this challenging task.

Finally, we test the model in a closed loop scenario on a task defined in continuous space and time with sparse rewards: the Mountain Car problem provided by the OpenAI Gym (Brockman et al., 2016). Once again, the clustered network with plastic input projections learns the task more effectively than an unclustered network or one with static input projections.

Notably, the network model is configured almost identically for these two quite dissimilar tasks, differing only in the mechanism by which the reinforcement learning signal is generated. In particular, the number of clusters is not optimized for the task, and the initial mapping of inputs to the representation layer is random. We thus conclude that the three components of our network model, namely, unsupervised learning on the input projections, clustered connectivity in the representation layer, and reinforcement learning on the output projection, combine to create a system possessing substantial generic learning capacity, requiring neither previous knowledge on how to partition the input space, nor training with biologically unrealistic supervised approaches. We anticipate that the components of unsupervised learning and clustered connectivity could also be employed to amplify the performance of other learning network models.

## 2. Methods

### 2.1. Network Architecture

**Figure 1:**
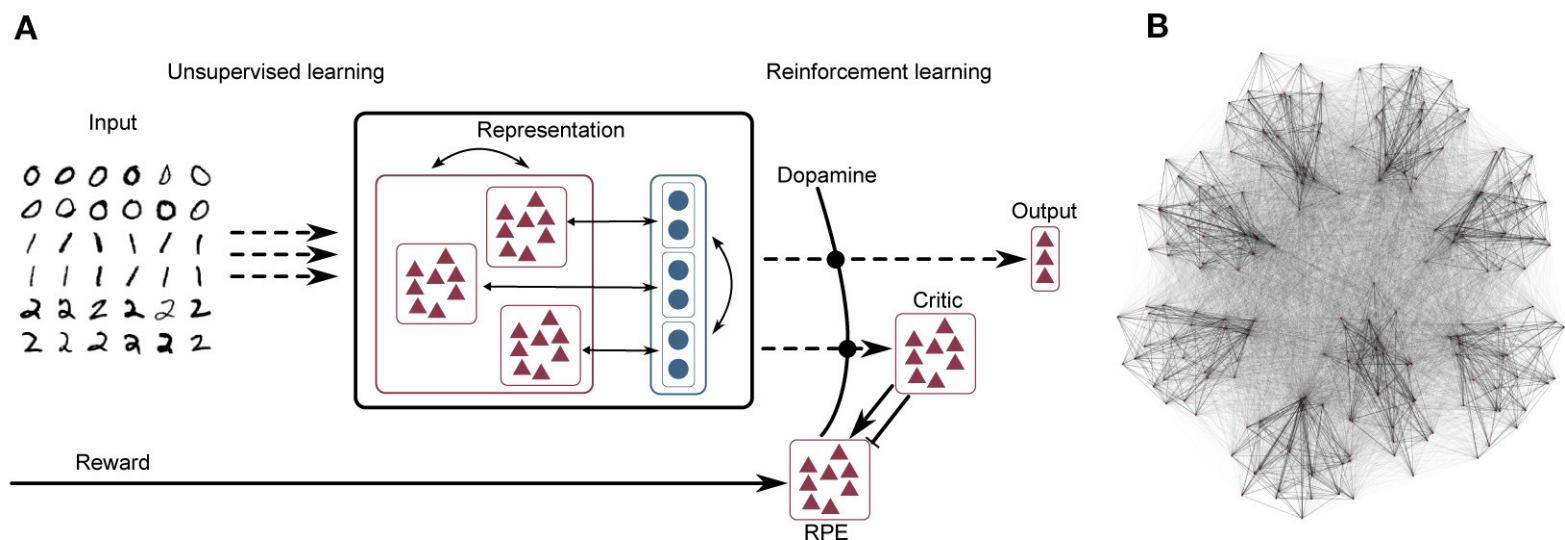
**(A)** Network schematic. Dashed lines represent plastic synapses. Synapses between the input layer and the representation layer are trained in an unsupervised fashion, synapses to the critic and readout are trained with reinforcement learning. **(B)** Visualization of network connectivity of a subset of the clustered balanced random network.

The network model consists of three layers, as illustrated schematically in Figure 1A. A complete tabular specification of the network and its parameters can be found in the Supplementary Materials.

#### Input Layer

The input layer is a population of rate modulated Poissonian spiking neurons with a maximum firing rate of *F*_max_. It converts the analog input data into spike trains for the representation layer. For example, a grayscale image can be presented to the network by stimulating each input neuron with the intensity *g*_*i*_ of a specific pixel in the image, resulting in Poissonian spike trains with a rate $f_{i}=F_{\text{max}}\cdot \frac{g_{i}}{255}$. See section 2.3 for details on the input conversion of each task.

#### Representation Layer

The input layer projects to the representation layer, consisting of 5, 000 integrate-and-fire neurons of which 4, 000 are excitatory and 1, 000 are inhibitory. The neuron types are largely identically parameterized, except for the membrane time constant τ_m_ and the applied bias current *I*_bias_; see the Supplementary Materials for a complete listing of the parameters. The structure of the representation layer is a balanced random clustered network, as described in Rost et al. (2018) and Rostami et al. (2020): the connection probability is uniform, but synaptic weights within a cluster (containing both excitatory and inhibitory neurons) are scaled up while the weights between clusters are scaled down, such that the total synaptic strength remains constant (see Figure 1B for a visualization of the network). The membrane dynamics follow the following equation:

(1) $$\begin{array}{r} \frac{dV}{dt}=\frac{-V\left(t\right)}{\tau_{\text{m}}}+\frac{I\left(t\right)}{C} \end{array}$$

The input current *I* is composed of the synaptic inputs from the input layer and from recurrent connections, and a bias current which is constant, and the same for all neurons of the same type (excitatory/inhibitory)

(2) $$\begin{array}{r} I\left(t\right)=\underset{i\in N_{\text{inp}}}{\sum }I_{i}\left(t\right)+\underset{j\in N_{\text{rep}}}{\sum }I_{j}\left(t\right)+I_{\text{bias}} \end{array}$$

where *N*_inp_ is the number of input neurons and *N*_rep_ the number of neurons in the representation layer. Each spike arriving at a synapse evokes an exponential-shaped post-synaptic current, so the input current due to the activity of one pre-synaptic input *i* is given by

(3) $$\begin{array}{r} \frac{dI_{i}}{dt}=\frac{-I_{i}\left(t\right)}{\tau_{\text{s}}}+\underset{t_{i}^{f}<t}{\sum }w_{i}\left(t\right)\delta \left(t-t_{i}^{f}\right) \end{array}$$

where τ_s_ is the relaxation time of the exponential post-synaptic current, $t_{i}^{f}$ is the set of spike times of neuron *i*, *w*_*i*_ the strength of the connecting synapse, and δ the Dirac delta function.

#### Output Layer

The output layer consists of integrate-and-fire neurons which follow the same dynamics as in Equation (1). The output neurons form a winner-takes-all circuit with lateral inhibition.

(4) $$\begin{array}{r} I\left(t\right)=\underset{j\in N_{\text{rep}}}{\sum }I_{j}\left(t\right)+\underset{k\in N_{\text{out}}}{\sum }I_{k}\left(t\right)+I_{\text{bg}}\left(t\right) \end{array}$$

where *N*_out_ is the number of output neurons, *I*_bg_ is background input from a Poisson spiking process, with current dynamics for individual synapses given by Equation (3). Analogously to the neurons of the representation layer, the feed-forward connections are plastic, with dynamics described below, while the recurrent inhibitory connections and the connections from the Poisson source are static. The number of output neurons corresponds to the number of labels in the dataset (for classification tasks) or the number of actions (for reinforcement learning tasks with discrete actions). Due to the strong lateral inhibition, at any given point in time, only one output neuron is strongly active, namely the neuron with the highest weighted input.

To determine which label (or action) is to be selected, the spikes of the neurons in the output layer are low-pass filtered with an exponential kernel; the predicted label (or chosen action) is then defined as that label (or action) associated with the output neuron with the highest activity.

#### Actor-Critic Circuit

The actor-critic approach is commonly used in reinforcement learning tasks where an immediate reinforcement signal is not available (Sutton and Barto, 2018). In this framework, the actor selects the actions, whilst the critic calculates the expected value *V*(*S*) (i.e., the discounted total expected future rewards) of the environment's states. From the difference between the value of the state before the action, and the sum of the reward received for an action and the discounted value of the new state, a reward prediction error (RPE) δ_*t*_ can be derived which expresses whether the action selected produced better or worse results than expected:

(5) $$\begin{array}{r} \delta_{t}=r_{t+1}+\gamma V\left(S_{t+1}\right)-V\left(S_{t}\right) \end{array}$$

where γ is the discounting factor. For values of γ close to zero, the agent is short-sighted and prefers immediate reward to rewards in the future. Values close to one correspond to a strong weighting of future rewards. This RPE is then used to update the expected value of the previous state and the policy of the agent, such that actions leading to better states than expected become more likely, and vice versa. In the context of neural activity, Frémaux et al. (2013) showed that a continuous signal, associated with the concentration of dopamine, can play the role of a reward prediction error:

(6) $$\begin{array}{r} D\left(t\right)=\overset{∙}{v}\left(t\right)+r\left(t\right)-\frac{1}{\tau_{r}}v\left(t\right) \end{array}$$

where *v* is the rate of a critic neuron (or population of neurons), *r* is the reward received directly from the environment and τ_*r*_ is the discounting time constant.

We implement the critic as a population of 20 Poissonian spiking neurons. The rate of this population is determined by which clusters are currently active, and the weights of the synapses connecting the neurons of the clusters to the critic neurons. For the purpose of establishing the necessary relationship for Equation (6), we interpret higher rates ν of the critic population as higher values *V*(*S*), in the sense of Equation (5), for the state associated with the active cluster (or clusters). The critic neurons project to a population of 1, 000 Poissonian spiking neurons representing the RPE, which in turn produce the dopaminergic concentration *D*(*t*) as described above. The instantaneous change of $\overset{∙}{v}\left(t\right)$ is implemented (as in Jordan et al. 2017) as a double connection from the critic to the RPE where one connection is excitatory with a small delay of 1 ms and the second is inhibitory with a larger delay of 20 ms (Potjans et al., 2009; Jitsev et al., 2012). Note that no claim is made for the biological plausibility of this circuit; it is simply a minimal circuit model that generates an adequate reward prediction error to enable the investigation of the role of clustered structure in generating useful representations for reinforcement learning tasks. The RPE signal enters the plasticity of the synapses between the representation layer and the output layer (i.e., the actor) as a third factor, as described in the next section.

### 2.2. Plasticity

The recurrent synapses within the representation layer are static; learning in this network model is carried out according to an unsupervised rule at the input projections (i.e., between the input layer and the representation layer) and according to a reinforcement learning rule at the output projections (i.e., between the representation layer and the output layer). This segregation between purely unsupervised learning in the input projections vs. reinforcement learning in the output projections is illustrated in Figure 1A.

The projections from a pre-synaptic neuron *i* in the input layer to a post-synaptic neuron *j* in the representation layer are subject to an unsupervised, local plasticity rule, as proposed by Tetzlaff et al. (2013):

(7) $$\begin{array}{r} \Delta w_{ji}=\mu \left(F_{i}F_{j}+\kappa \left(F^{T}-F_{j}\right)w_{ji}^{2}\right) \end{array}$$

Here, the parameter μ sets the global learning rate, whereas κ controls the ratio of synaptic scaling relative to Hebbian modifications. The firing rates of the pre- and post-synaptic neurons (*F*_*i*_ and *F*_*j*_, respectively) are calculated by low-pass filtering the spike trains with an exponential kernel with a fixed time constant τ = 100 ms; *F*^*T*^ constitutes the *homeostatic set point*, i.e., the target firing rate.

The learning rule comprises a Hebbian component, dependent on pre- and post-synaptic activity, and a non-Hebbian, homeostatic term with a quadratic weight dependence (see Tetzlaff et al., 2013 and references therein). While the Hebbian term establishes an appropriate, input-specific mapping, the homeostatic term guarantees convergence and stability in the resulting weights, while dynamically retaining their integrity (relative weight distribution). The addition of weight regularization as a homeostatic mechanism is a biologically-compatible procedure to control weight growth and the stability of learning and population activity (see e.g., Tetzlaff et al., 2011, 2012; Yger and Gilson, 2015). Importantly, when applied to simpler rate models, quadratic weight regularization converges onto the eigenvectors of a neuron's input covariance matrix, transforming a simple rate neuron in a principal component analyzer (see Oja, 1982).

For the synapses between a pre-synaptic neuron *i* in the representation layer and a post-synaptic neuron *j* in the output layer, we extend the rule given in Equation (7) to incorporate an explicit reinforcement signal (making it a three-factor learning rule). Specifically, the Hebbian term is complemented with a multiplicative modulatory term. Learning in the output projections thus takes the form:

(8) $$\begin{array}{r} \Delta w_{ji}=\mu \left(\left(D-b_{D}\right)F_{i}F_{j}+\kappa \left(F^{T}-F_{j}\right)w_{ji}^{2}\right) \end{array}$$

The reinforcement signal is modeled as the dopaminergic concentration *D* relative to its baseline value *b*_*D*_, which in turn is computed as a moving average over the last 10 s.

### 2.3. Tasks

#### 2.3.1. Logical Operations Task

We chose four different logical operations (see Table 1) to test our setup; OR, AND, XOR and “material implication” (IMPL). Three of the operations are linearly separable (OR, AND, IMPL) whereas XOR is not.

**Table 1:** Truth table of the logical operations.

**A**	**B**	**OR**	**AND**	**XOR**	**IMPL**
0	0	0	0	0	1
0	1	1	0	1	1
1	0	1	0	1	0
1	1	1	1	0	1

The input neurons (stim A and stim B) can either be “on” or “off,” where “on” means that the neuron is highly active (1, 000 Hz firing rate) and “off” means nearly silent (10 Hz firing rate). Each possible input combination is presented over a 500 millisecond period, which is repeated for the entire simulation. We record 50 s of low-pass filtered neural activity (1, 000 Hz sampling frequency) in the absence of plasticity, then apply the Tetzlaff rule (Equation 7) to the input projections for the rest of the simulation (500 s in total). The filtered spiking activity is once again recorded between 450 and 500 s.

The recordings were used to train an ordinary least square linear regressor (Table 1). Each stimulus contains 500 samples which are trained against the values of the truth table. For example, in the XOR task, each sample of input (1, 1) should yield “0.” In order to determine the accuracy of the regressor, we use a threshold of 0.5, i.e., values greater or equal to 0.5 are considered correct if the target value is “1,” and values less than 0.5 are considered correct if the target value is “0.”

#### 2.3.2. MNIST Task

MNIST^1^ is a dataset of handwritten digits from zero to nine. Each digit is presented as 28 × 28 pixel grayscale images and the whole dataset contains 60, 000 training images and 10, 000 test images. As the runtime of our network is too slow to present the complete dataset, we use only the first three digits. We further reduce the 18, 000 training and 3, 000 test images to 1, 000 randomly picked images for the training phase and 150 for the test phase.

For each trial during the training phase, one digit was picked randomly and presented to the network. The images are translated into spiking activity using one neuron per pixel as described in section 4. The dopaminergic signal *D*, which enters the plasticity equation Equation 8 as a third factor, is generated using a neuron which doubles its base firing rate for 100 ms if the label predicted by the network is correct (see *Output Layer* in section 2.1), and becomes silent for 100 ms if it is incorrect. As such, the dopaminergic signal is equivalent to an immediate positive or negative reward *r* for success or failure. Note that the correct/incorrect feedback, rather than the identification of the correct class, renders this a reinforcement learning task rather than a supervised task.

#### 2.3.3. Mountain Car Task

**Figure 2:**
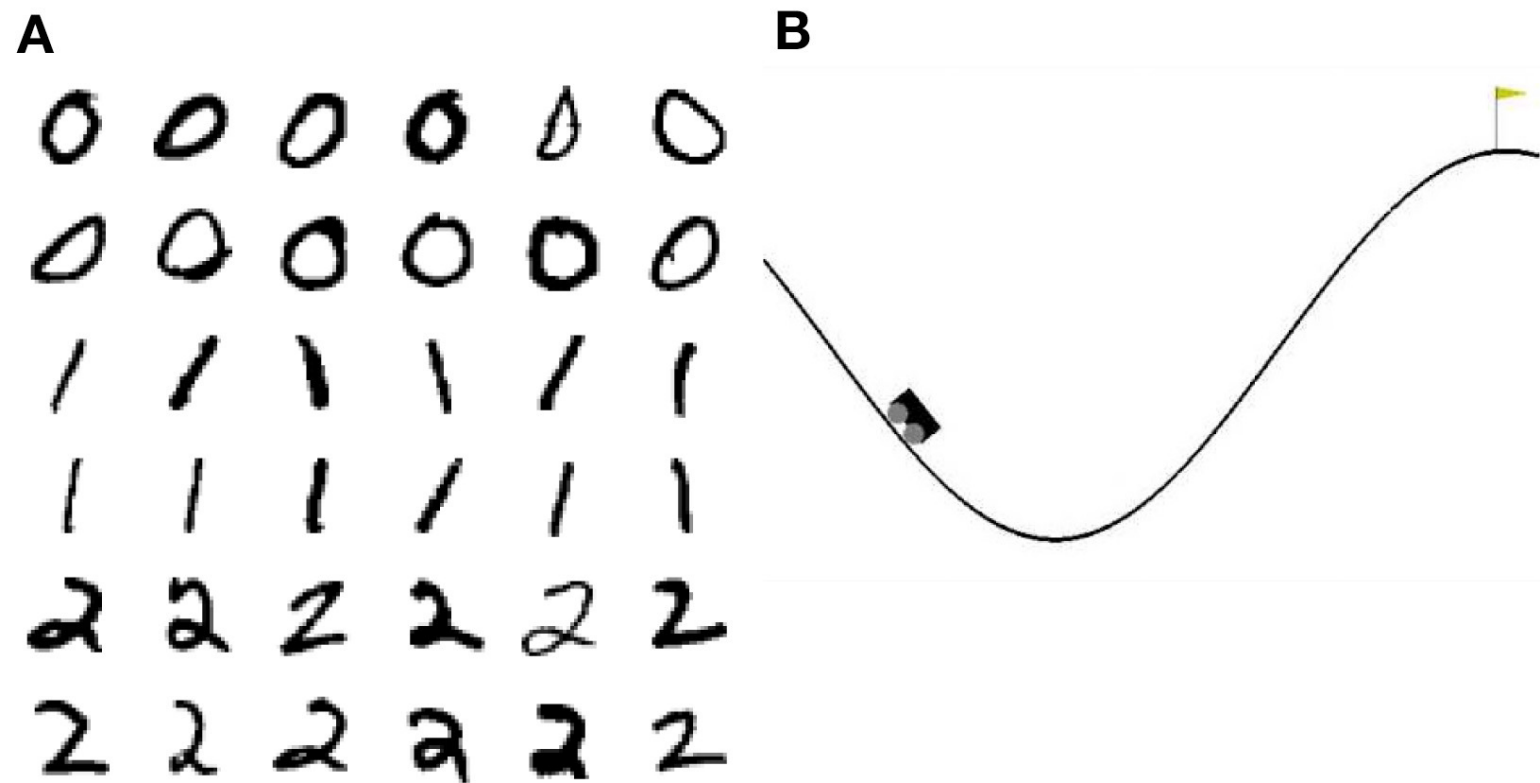
Visualization of the tasks. **(A)** 36 example images of the 3-digit subset of the MNIST database. **(B)** Visualization of the Mountain Car environment of the OpenAI Gym.

Mountain Car^2^ is a reinforcement learning environment of the OpenAI Gym. In this task, a car is randomly placed between two hills. The goal is to reach the top of the right hand hill. This task is difficult because the engine of the car is not strong enough to just drive up the hill in one go; instead the agent must build up momentum by swinging back and forth until the car finally reaches the top (see Figure 2B).

The state space of the Mountain Car task is continuous, consisting of the (real-valued) velocity and position of the car, while the action space is discrete. The three discrete actions are “left,” “right” and “nothing” which alter the velocity of the car. If actions “left” or “right” are chosen, the velocity changes by a fixed value in positive (“right”) or negative (“left”) direction. Additionally, the velocity is also affected by gravity, meaning that the car automatically rolls downhill if action “nothing” is chosen. The position of the car changes in each timestep according to its velocity.

The available information for the agent consists only of the position and the velocity of the car and, in every timestep, the agent receives a constant punishment of −1. The episode terminates, and the environment resets, if the car reaches the goal position or a limit of 200 timesteps is reached.

We adapted the environment in two minor ways. First, we removed the time limit of 200 timesteps in order to enable our agent to explore the environment. Second, we added a positive reward of 1 when the car reached the goal position to give the reinforcement learning algorithm a higher contrast to the constant punishment during the trials.

The OpenAI Gym defines the task as solved if less than 110 timesteps are needed to reach the goal for 100 consecutively trials. This is not easy to achieve as the car starts in a random position between the hills and in some cases it is necessary to first swing to the right, then to the left and finally to the right again. From the worst starting position the timing must be perfect to solve the task within 110 timesteps.

The OpenAI Gym defines the task with a position *x* ∈ [−1.2, 0.6] and velocity *ẋ* ∈ [−0.7, 0.7] (arbitrary units) which we normalize to [−1, 1] for convenience. The real-valued, two-dimensional signal is then converted into a spiking signal by the input layer, consisting an array of 200 neurons for each dimension, where the activation *a*_*i*_(*t*) of the *i*th neuron along each dimension is determined by a Gaussian shaped receptive field of mean $\mu_{i}=-1+\frac{2i}{N}$ and sigma σ_*i*_ = 0.05. On the basis of these activations, the input layer neurons emit Poissonian spike trains with firing rates given by 35*a*_*i*_(*t*), i.e., between 0 and 35spks/s.

The readout consists of three neurons representing the three different actions “left,” “right,” and “nothing.” As in the other tasks, the readout neurons are in competition, ensuring that they are not active at the same time.

### 2.4. Simulation Tools

All neural network simulations were performed using the Neural Simulation Tool 2.16 (NEST) (Gewaltig and Diesmann, 2007; Linssen et al., 2018). The interface between NEST and the OpenAI Gym (Brockman et al., 2016) was implemented using the ROS-MUSIC Toolchain (Weidel et al., 2016; Jordan et al., 2017). Simulation scripts and model definitions are publicly available^3^.

## 3. Results

### 3.1. Learning Input Representations and Resolving Non-linearities

**Figure 3:**
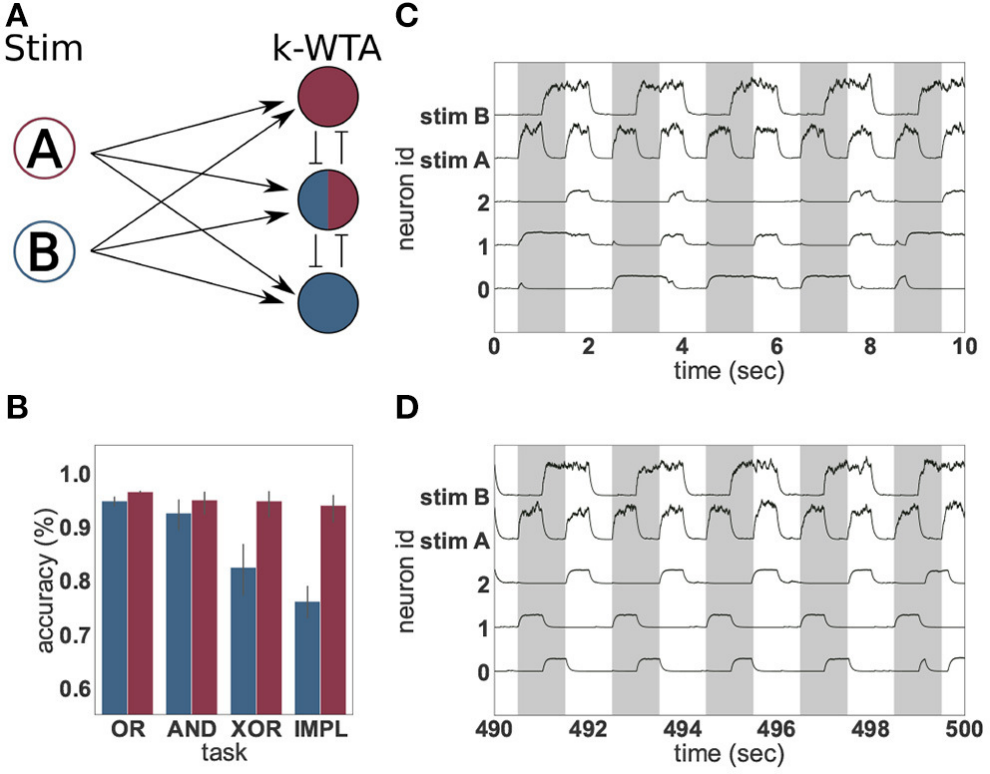
Solving logical tasks with unsupervised learning. **(A)** Network schematic of a two-layered network comprising two stimulation neurons (A and B) and three representation neurons implementing a k-winners-take-all architecture. **(B)** Accuracy (%) of linear regression on 50 s of low-pass filtered activity of the representation neurons before (blue) and after (red) unsupervised learning was applied to the input projections. Four different logical tasks were used; OR, AND, XOR and implication (IMPL). The result was averaged over 30 trials with different random seeds, the error bars indicate the 95% confidence interval. **(C)** Low-pass filtered neural activity before unsupervised learning was applied. The gray bars indicate when the logical XOR task should yield “1.” **(D)** as in **(C)**, but after 440 s of unsupervised learning.

The computational benefits conferred by the ability to learn suitable input representations are clearly demonstrated by the simple example shown in Figure 3, employing the logical operations “or” (OR), “and” (AND), “exclusive-or” (XOR) and “material implication” (IMPL), of which XOR is the most fundamental non-linear task. Whereas the XOR task can be trivially solved by trained artificial neural networks (Gelenbe, 1989) and even by untrained recurrent networks of varying degrees of complexity (using the reservoir computing approach, e.g., Haeusler and Maass 2007; Verstraeten et al. 2010; Zajzon et al. 2019), the combination of a small network size and random input projections can result in an inadequate transformation that doesn't allow the non-linear task to be resolved. On the other hand, randomness in the input projections can give rise to heterogeneity in response selectivity, which may suffice to resolve the non-linear separability problem, provided no additional sources of response variability are introduced in the representation neurons (Rigotti et al., 2010). However, the application of an appropriate unsupervised learning rule allows even a very small and simple network of spiking neurons to resolve non-linearities in the input, by adapting the projection of the input signal onto the neuronal representational space to develop clear stimulus representations.

To demonstrate, we define an *input layer* comprising two neurons (A and B) which represent “1” and “0” by being either highly active or nearly silent, respectively. The activity of these input neurons is randomly projected to a higher-dimensional space spanned by three integrate-and-fire (IAF) neurons forming a weak winner-take-all network, the *representation layer*. The projections from the input to the representation layer (weights initialized randomly from a uniform distribution between 33.33 and 66.66) are subject to an unsupervised, local plasticity rule with Hebbian and homeostatic components (see Equation 7 and Tetzlaff et al., 2013). The Hebbian part of the plasticity rule strengthens the weights between stimuli and active neurons and increases the probability that the same input evokes the activation of the same neurons. The scaling part introduces competition between the neurons and ensures that an active neuron does not become active for all stimuli. Linear regressors are trained on the activity of the representation neurons to approximate the four logical operations. This simple setup is illustrated in Figure 3A and described in greater detail in section 2.3.1).

As Figure 3B shows, the presence of the unsupervised plasticity supports the reliable formation of stimulus-tuned neurons. A linear regressor trained on the first 50 s of the activity of the representation neurons, before plasticity is switched on, is able to learn the simple linear tasks (OR and AND), but shows significantly worse performance in the case of IMPL and the non-linear task XOR. This is due to the activity of the representation layer (Figure 3C), which is irregular and lacks adequate input specificity to resolve the non-linear task—none of the neurons are reliably “on” or “off” for either the case where XOR should resolve to “1,” or where it should resolve to “0.”

In contrast, after the input projections have been adapted by the unsupervised learning rule, the representation neurons exhibit a reproducible and input-specific response, as shown in Figure 3D: neurons 1 and 2 have a high rate, and neuron 0 a low rate, when XOR should resolve to “1,” and vice versa when it should resolve to “0.” Consequently, the linear regressor trained on the last 50 s of neural activity, shows a good performance for all four logical operations. Unsupervised learning on the input projections thus yields significant gains by enforcing response specificity in the representation layer.

As discussed in section 2.2, the use of weight regularization as a homeostatic mechanism (Equation 7) has long been known to play an important role in feature acquisition (Oja, 1982; Tetzlaff et al., 2011, 2012, 2013; Yger and Gilson, 2015). Thus, these results are unsurprising in themselves, but serve as a demonstration that unsupervised learning introduces important specificities into the population dynamics that allow it to be used as a substrate for further computation, as we will show in the following sections.

### 3.2. Feature Extraction in a Classification Task

In the previous section, we demonstrated that the unsupervised learning rule we implement is capable of learning input representations that enable a simple system to resolve non-linearities in the input signal. To further examine its capabilities, we now explore a more challenging task and a more complex system. The task we explore in this section is handwritten digit recognition (the MNIST dataset), most commonly used for image classification, for which the algorithm has to detect handwritten digits in grayscale images of size 28 × 28. Due to the high computational load for the numerical simulations, we reduce the full dataset to a three digit subset. Moreover, we limit the training set to 1, 000 images and the test set to 150 images, which is just a small fraction of the available data. Details of the experimental set-up are given in section 2.3.2.

The network structure is illustrated schematically in Figure 1A and described in detail in section 2.1. As for the previous task, the network we implement consists of an input and representation layer, but is now supplemented with an *output layer*, which is realized by a soft winner-takes-all circuit of three integrate-and-fire neurons, one for each label in the dataset; the most active neuron is interpreted as the network's decision on which label corresponds to its current input.

As the input dimensionality is much larger in the MNIST task than in the logical XOR task, a larger representation layer is needed, which we implement as a clustered balanced network as described by Rost et al. (2018) and Rostami et al. (2020). In this model, all pairs of neurons have the same connection probability, but for neurons defined as belonging to the same cluster, the connection weight is increased (with respect to the reference weight of the corresponding unclustered network). All other weights are decreased to compensate, so each neuron receives the same total weighted synaptic input. This is visualized in Figure 1B. Unlike the clustered network architecture investigated by Litwin-Kumar and Doiron (2012), we cluster both excitatory and inhibitory connectivity. Rost et al. (2018) showed that networks comprising purely excitatory clusters tend to fire at saturation in the active clusters, with very low activity elsewhere, resulting in only infrequent switches between active clusters. By introducing inhibitory clusters, the firing rate of the active cluster does not saturate, which facilitates cluster switching.

For the purposes of comparison, we consider both a network parameterized to have eight clusters, such that the competition between the clusters is very strong and a switch between the winning clusters is rare, and an equivalent balanced network with random connectivity, i.e., no clusters. The total amount of excitation and inhibition in both networks are identical, which is reflected in their identical average firing-rate of ~27 spikes per second. Dynamically, the difference can be measured using the coefficient of variation (CV) of the inter-spike-intervals. The average CV of the unclustered network is 0.92, which is close to the expected value of a Poisson process. In contrast, the neuronal activity in the clustered network varies much more (CV 4.61) due to the fluctuating activity of the clusters.

The synapses between the input and representation layers evolve according to an unsupervised local learning rule as for the XOR task, and the synapses between the representation and output layers adapt according to an unsupervised rule with an additional neuromodulatory multiplicative term, see Equations 7 and 8. Thus, as illustrated in Figure 1A, the input projections are subject to purely unsupervised learning, whereas the output projections, due to the neuromodulatory third factor, are subject to reinforcement learning.

Note, firstly, that the reinforcement learning signal presented to the output projections denotes only success or failure (see section 2.3.2) and is therefore less informative than the supervisory learning signal usually used for this task (which would denote the correct choice in case of failure), and secondly, that there is no error back-propagation from the output layer to the representation layer to help solve the credit assignment problem. Thus, the classification performance depends on a stable and consistent representation of the input.

#### 3.2.1. Self-Organization of Feature Extracting Representations

**Figure 4:**
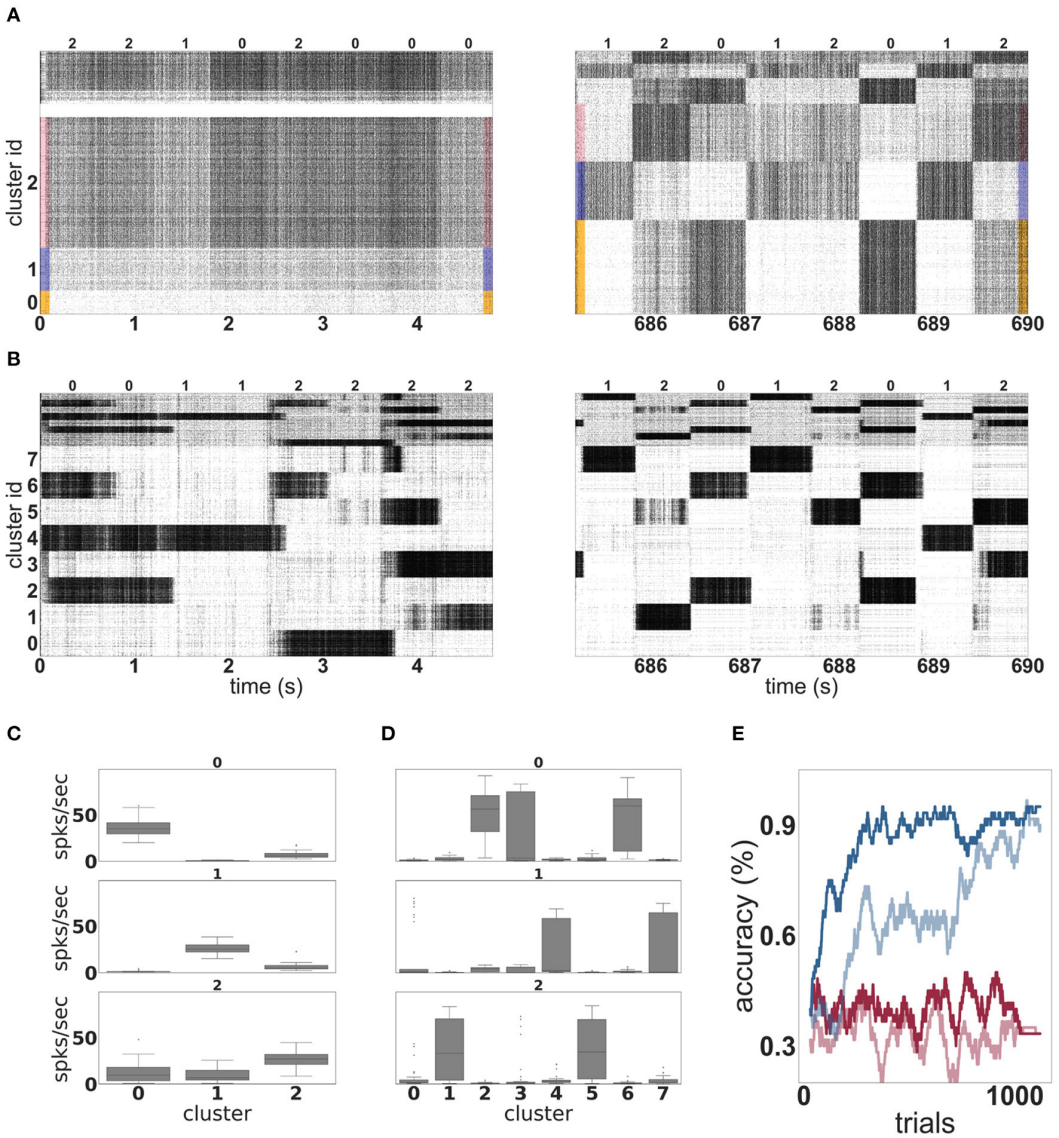
Dynamics and performance of the model during the MNIST task. **(A)** Raster plot of an unclustered balanced random network model during the first and the last 5 s of the simulation while solving the MNIST task. The identity of the stimulus digit is shown above the plots. The first 4, 000 neurons are excitatory, and the last 1, 000 are inhibitory. Some neurons are silent in the beginning of the simulation. **(B)** As in **(A)**, but for a network with eight clusters. **(C)** Activation of the three emergent clusters in the initially unclustered network during the presentation of the last 100 stimuli (indicated above the panels). The boxes cover the interquartile range (IQR) of the corresponding cluster activations. The whiskers extend to the values within 1.5 times the IQR. **(D)** As in **(C)** but for the network with eight clusters. **(E)** Classification performance of the unclustered (pale colors) and clustered (dark colors) networks (one instance each). Red traces indicate the performance of the corresponding networks without unsupervised plasticity on the input projections, whereas blue traces correspond to networks with plastic input synapses. Performance is calculated using a sliding average over a window of 30 trials.

Figure 4A shows the evolution of spiking activity in the unclustered network for one trial of the MNIST task. The neurons are ordered by their maximal response to the three different input classes; the excitatory neurons most responsive to stimuli of class “*zero”* are at the bottom of the plot, then those most responsive to digit “*one,”* followed by digit “*two.”* This ordering is repeated at the top of the plot for the inhibitory neurons. In the first 5 s (left panel), no clear distinction can be made between the activity of the network on the basis of the input digit, although some overall changes in firing rate can sometimes be observed between two input stimuli. The white horizontal bar in the plot is due to neurons that do not fire at all during the recording period. However, by the last 5 s (right panel), there are easily discernible differences in the spiking activity corresponding to the individual input digits.

Through the action of unsupervised learning in the input projections, the network has self-organized into effective clusters—effective in the sense that only the input weights have adjusted, not the recurrent weights—that represent the digits “*zero,” “one,”* and “*two.”* Whereas, the modifications introduced by this learning rule when applied to recurrent synapses have been shown to lead to the acquisition and formation of stable cell assemblies (Auth et al., 2020), our results demonstrate that applying it to the input projections alone can yield the development of feature specialization. Coincident input and representation neurons' activations, elicited by the pattern of external stimuli, leads to the strengthening of specific pathways. Learning is stabilized and distributed such that the input projections “compete” to establish sub-population specific responses in the representation layer, ensuring the formation of lasting input traces. These results are in line with Tetzlaff et al. (2012), where a similar learning rule lead to the formation of input-specific pathways in a simple feed-forward architecture, through a stable sequence of fixed points.

The effect of this self-organization on the network dynamics is most evident in the coefficient of variation of the spike trains, which increases from 0.9 at the beginning of the trial to 2.7 at the end of the trial. The average firing rate is hardly changed, dropping from 27 to 26 spikes per second.

In contrast to the unclustered network, the activity in the clustered network is already strongly differentiated in the first 5 s (see Figure 4B, left panel). The clusters switch on and off at the transition points between stimuli. At this point, it is hard to discern stimulus-specific patterns—for example, cluster 0 is sometimes on for a presentation of the digit “*two,”* and sometimes off. By the end of the trial (right panel), the activity has coalesced into clear stereotypical patterns: as an example, stimuli from the class “*one”* always evoke activity in clusters 4 or 7. The change in the network response to stimuli is less visible on the level of spike train statistics than that of the unclustered network; the CV increases from 4.6 to 5.2 and the average firing rate decreases from 27 to 25 spikes per second.

The detailed response profiles for each stimulus class can be seen in Figures 4C,D. Notably, while each stimulus can be represented by more than one cluster, there is no overlap between the profiles—each cluster responds to only one stimulus class.

The performances of the two network configurations are shown in Figure 4E. In the absence of unsupervised plasticity at the synapses between the input and the representation layer, both the unclustered and the clustered network perform at chance level. In the presence of plasticity, both networks reach a good performance, clearly demonstrating the importance of learning input projections for this task. Of the two networks, the clustered network performs consistently better—it learns faster, reaching an accuracy level of 80% after 181 trials and 90% after 268 trials, compared to 786 and 1, 014 for the unclustered network. The performance of the clustered network is also better after saturation than that of the unclustered network. Over the last 100 trials, the clustered network has an average of 96%, compared with 92% for the unclustered network.

To acquire some insight into why the clustered network performs better, we consider the features of the input data and their representation in the network. The three digits selected from MNIST have a large overlap and are not linearly separable, similar to the XOR task examined in section 3.1. However, in contrast to XOR, the classes in MNIST have a large intra-class variance: there are sub-classes of different styles of writing the digits. To give some examples, there is a sub-class of the digit “*zero”* written close to circular while other sub-classes are more tilted or oval; the digit “*one”* is often written as vertical line, but sometimes also tilted; the digit “*two”* may be written with or without a loop. A sample of these varying styles can be seen in Figure 2A. This intra-class variability is part of what makes the MNIST task challenging. In order to solve the task, a classifier needs to learn a category that is broad enough to detect all the instances of a particular digit, whilst simultaneously being narrow enough to exclude all the instances of other digits.

**Figure 5:**
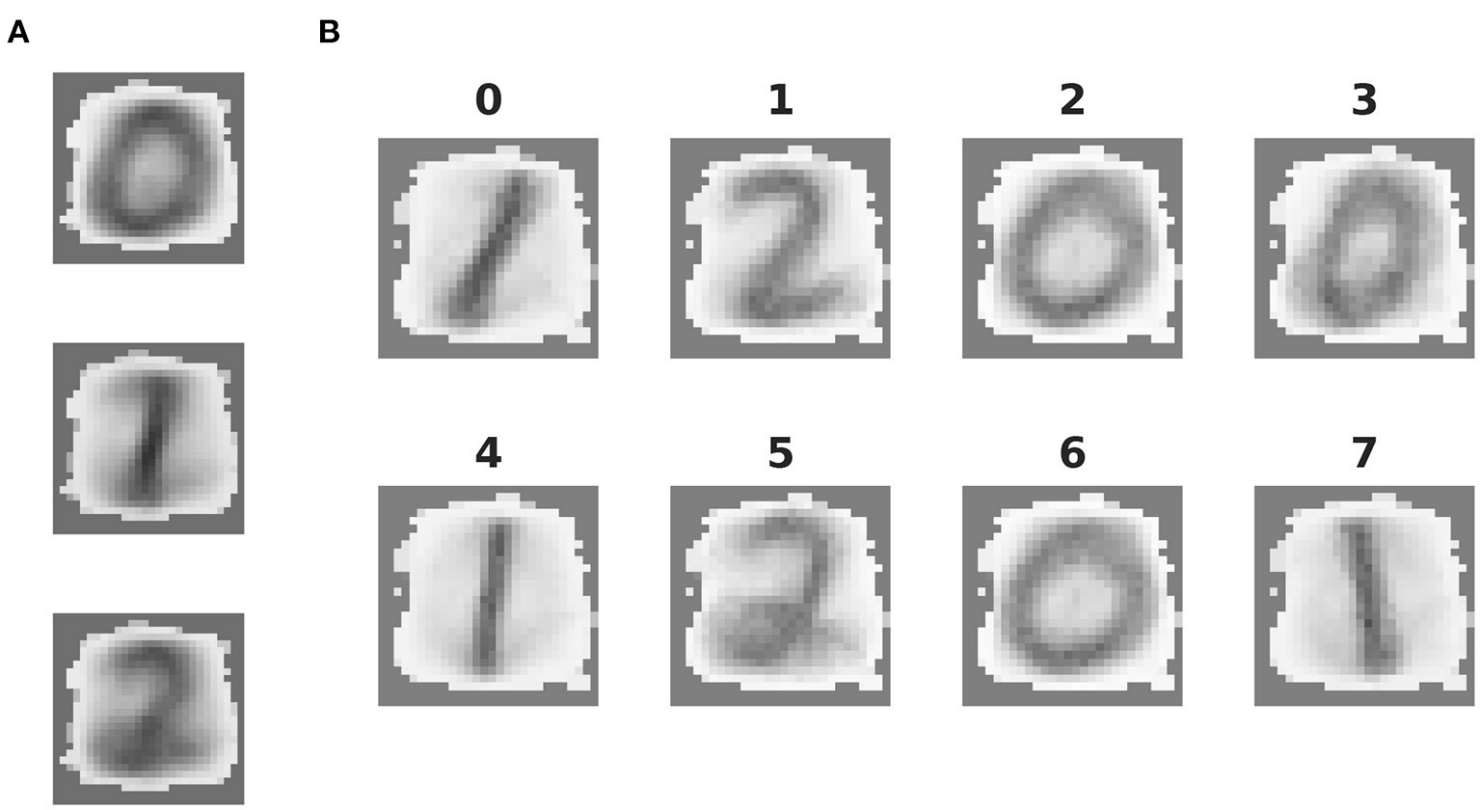
Features extracted by unsupervised learning. **(A)** Mean synaptic weights between the 28 × 28 input neurons and the three effective clusters of the initially unclustered network, see Figure 4A for the corresponding activity. **(B)** As in **(A)**, but for the eight clusters of the clustered network (cf. Figure 4B).

The mean synaptic weights between the input neurons and the neurons in each (effective) cluster of the representation layer correspond to the receptive field of each cluster, and thus the specialization learned in an unsupervised fashion by that cluster. These can be visually compared as in Figure 5. The receptive fields of the effective clusters that self-organize in the unclustered network are generic for each digit (Figure 5A). This results in a blurred appearance, because they contain traces of all the sub-classes of a particular digit. In contrast, the receptive fields of the clustered network have a less blurred appearance and reveal that each cluster has typically specialized itself for a specific sub-class. Thus, there are different clusters representing a round or oval “*zero,”* a straight or tilted “*one,”* and a looped or unlooped “*two.”* This specialization explains the pattern of activity observed in Figure 4B, right panel: different sub-classes of a digit evoke activity in different clusters. In addition, this explains the wide variance of the response profiles shown in Figure 4D and the performance shown in Figure 4E: the more specific receptive fields learned by the clustered network allow a stronger differentiation of a cluster's response to its preferred digit over non-preferred digits than the generic receptive fields of the unclustered network, leading to a higher accuracy.

**Figure 6:**
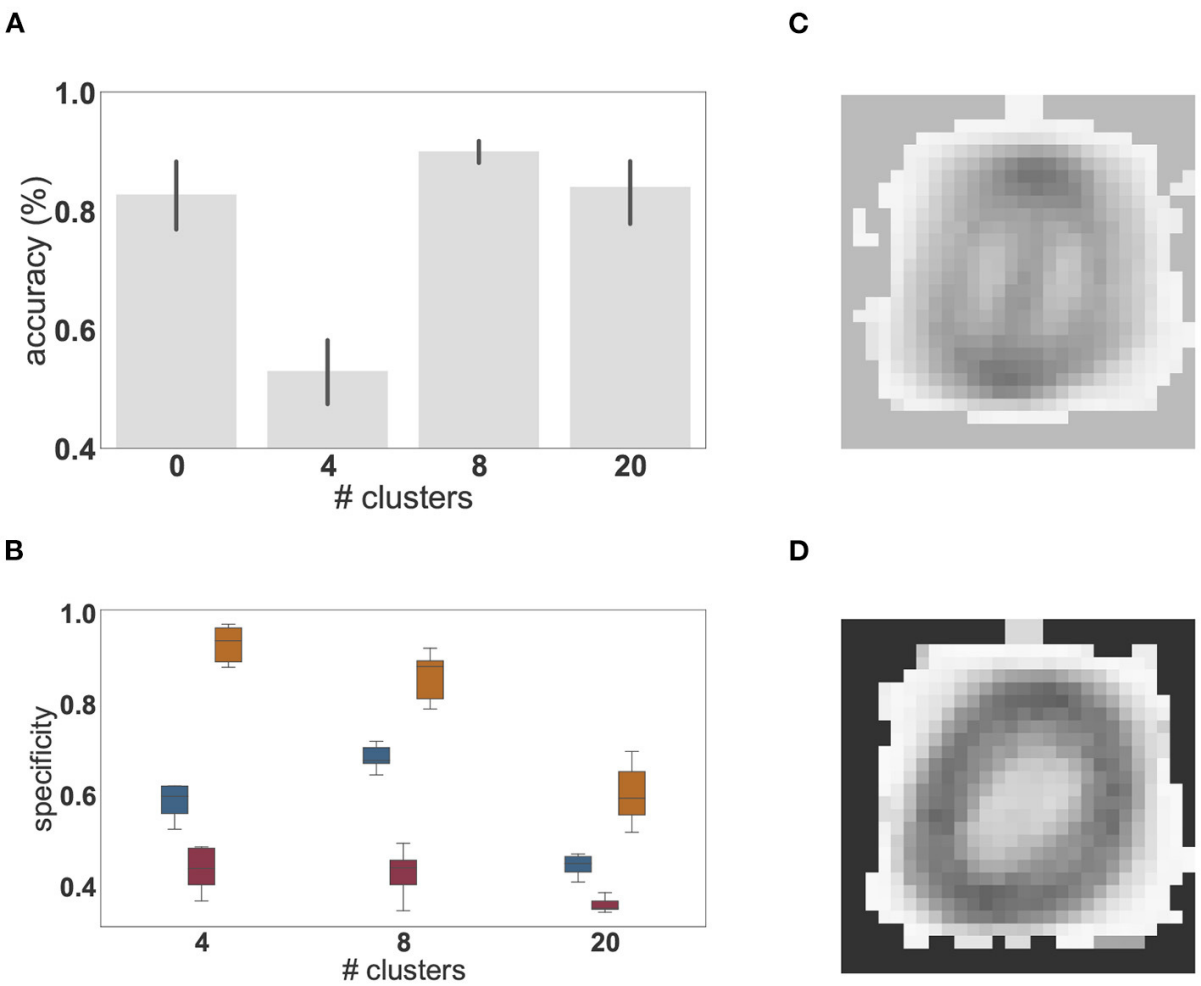
Dependence of performance and specialization on the number of clusters. **(A)** Classification accuracy (%) of linear regression on the low-pass filtered population activity for a random network (0 clusters) and networks composed of 4, 8, and 20 clusters on the MNIST task. Results were averaged over 10 network instantiations per condition; error bars indicate the 95% confidence intervals. **(B)** Distribution of mean (blue), minimal (red), and maximal (orange) cluster specificity across 10 network instantiations for networks with different numbers of clusters. The box plots cover the interquartile range (IQR), with whiskers extending to the values within 1.5 times the IQR. **(C)** Example of the extracted feature of a cluster with low specificity (0.34). **(D)** Example of the extracted feature of a cluster with high specificity (0.86).

Whereas these results show that unsupervised cluster specialization allows different neuronal sub-populations to become tuned not only to the different classes, but also to intra-class variations in the MNIST digits, the number of internal clusters required to achieve this remains to be determined. To investigate this relationship, we systematically varied the network's internal structure and evaluated the impact of having smaller and larger numbers of clusters on the overall classification performance and the maximum, average and minimum specificity of the clusters, measured as the sharpness of the output projections, i.e., how much stronger (in relative magnitude) is the strongest output projection. This is calculated as $S_{c}=W_{o}^{*}/\underset{o}{\sum }\left({\bar{W}}_{oc}\right)$, where ${\bar{W}}_{oc}$ is the average synaptic strength from all neurons in cluster *c* to output neuron *o* and $W_{o}^{*}$ is the maximum of ${\bar{W}}_{oc}$ over all output neurons, i.e., $W_{o}^{*}=\underset{o}{\max}\{{\bar{W}}_{oc}\}$. The results are shown in Figure 6.

We find that the peak performance is achieved by eight clusters (97.3%), which also exhibits the smallest 95% confidence interval. If the number of clusters is too small (four cluster condition in Figures 6A,B), feature specialization is insufficient to accurately discriminate the classes, dramatically reducing overall classification accuracy below that achieved by a random, unclustered network (zero cluster condition). Interestingly, the maximum specificity is highest for four clusters, indicating that some clusters become highly specific for specific classes. However, the average specificity across the network is smaller than in a network composed of eight clusters. This has a significant impact on the ability to distinguish the different digits and accounts for the substantial reduction in classification accuracy. For illustration, the receptive fields of two clusters with high and low specificity are given in Figures 6C,D.

On the other hand, if the representation layer is endowed with more clusters than are needed to solve the task (20 cluster condition in Figure 6), representations become redundant and the degree to which each cluster is tuned to particular digits is greatly reduced (Figure 6B) leading to multiple clusters specializing for the same features. Nevertheless, given that all classes and intra-class variants can be represented when distributed across the twenty clusters, the classification accuracy is still high, but the benefits of modular recurrent connectivity in the representation layer are lost, as its performance is closer to that of an unclustered network than to that of a network with eight clusters (Figure 6A).

### 3.3. Continuous Time and Space and Delayed and Sparse Reward

So far we have shown that our model can resolve non-linearities in the input and extract and represent complex features in images using the XOR and the MNIST classification tasks. In both cases, to train the readout weights with reinforcement learning, the reward or punishment is applied directly after the end of the stimulation period. This is not a very representative scenario for real-world tasks, where it is necessary to make a sequence of decisions which typically involve a substantial delay period until the reward is received (Schultz, 2016; Tervo et al., 2016). Moreover, unlike XOR and MNIST, which constitute toy examples, real-world stimuli unfold continuously in both time and space. Foraging is a prime example of a real world task which has the properties of continuous state-space and delayed reward.

We therefore investigate the performance of our model in a more challenging task, continuous in time and space and providing only sparse and delayed reward at the end of a successful trial: the “Mountain Car” reinforcement learning environment provided by the OpenAI Gym. In this task, at the start of each trial the car is placed in the valley between two hills (see schematic example in Figure 2B). The trial ends when the task is solved, i.e., the agent reaches the top of the right-hand hill by swinging back and forth to gain momentum, or when a timeout of 200 timesteps has been reached (see section 2.3.3). The only information available to the learning agent is the continuous position and velocity of the car.

As the reward is only presented at the end of a trial, we include an actor-critic architecture in our network structure which generates a reward prediction error (RPE). This is illustrated schematically in Figure 1A and described in detail in section 2.1. The availability of a RPE allows a learning agent to project a sparse reward back to earlier (non-rewarded) states. However, doing so requires a stable representation of environmental states. Whereas, previous demonstrations that networks of neurons can solve this task relied on hard-coded “place cell”-type representations of the environment (Frémaux et al., 2013; Jordan et al., 2017), our model self-organizes a representation of the environment that is adequate for learning the task.

**Figure 7:**
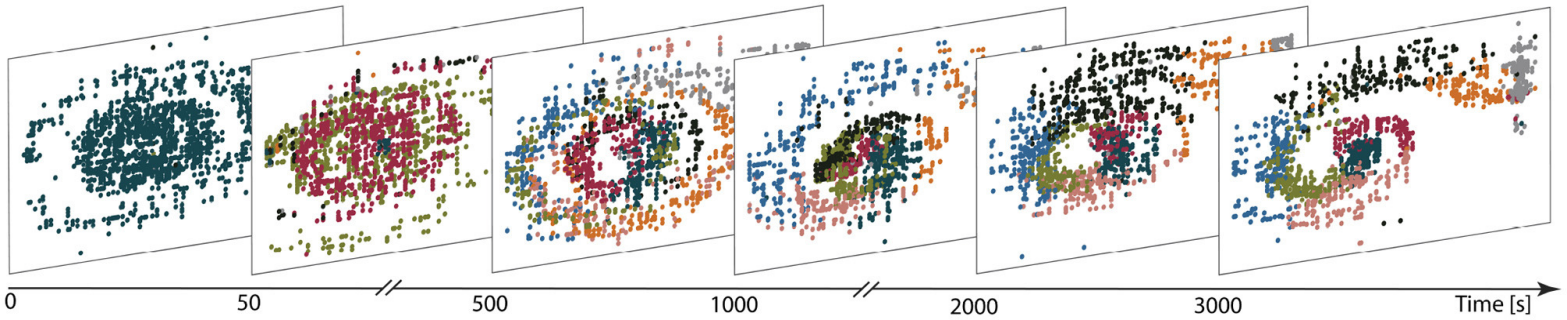
Learning functional partitions of the state space. Each panel depicts the cluster activations over a 10*s* window at the time shown on the figure axis, color-coded by cluster identity. Each dot represents the reconstructed position (panel horizontal axis) and velocity (vertical axis) of the car in the input space for 1 ms. The reconstruction is calculated on the basis of the spike trains filtered with an exponential kernel (τ = 30 ms); the neuron with the highest activity is assumed to reflect the current position/velocity of the car. Likewise, the most active cluster is defined to be the one with highest (filtered) activity in a given bin.

Figure 7 illustrates the evolution of the representation of the environment (in a network with eight clusters and plastic input projections) as the agent learns the task. Before training (first panel), a single cluster is active, independent of the agent's location in position-velocity space. Early in the learning process (after 50 s, second panel), other clusters are gradually activated and after 500 s (third panel), all neuronal clusters are actively firing. Some degree of specialization begins to emerge, whereby specific clusters are predominantly activated for specific regions of the task space. This differentiation gets sharpened as learning progresses, with some clusters substantially changing their preferred area of input space over time. For example, the black cluster is activated by a broad region of input space at *t* = 500 s, coalesces to the middle by *t* = 1, 000 s and then moves out to occupy the central upper region by *t* = 2000 s. The final partition exhibits a clear mapping from input space to cluster activation (3, 000 s; final panel). The final mapping demonstrates that, after exploring the environment for long enough, the clusters in the network become active for those parts of the state space which are frequently visited and therefore form a task-oriented, rather than comprehensive, discretization of the state space.

**Figure 8:**
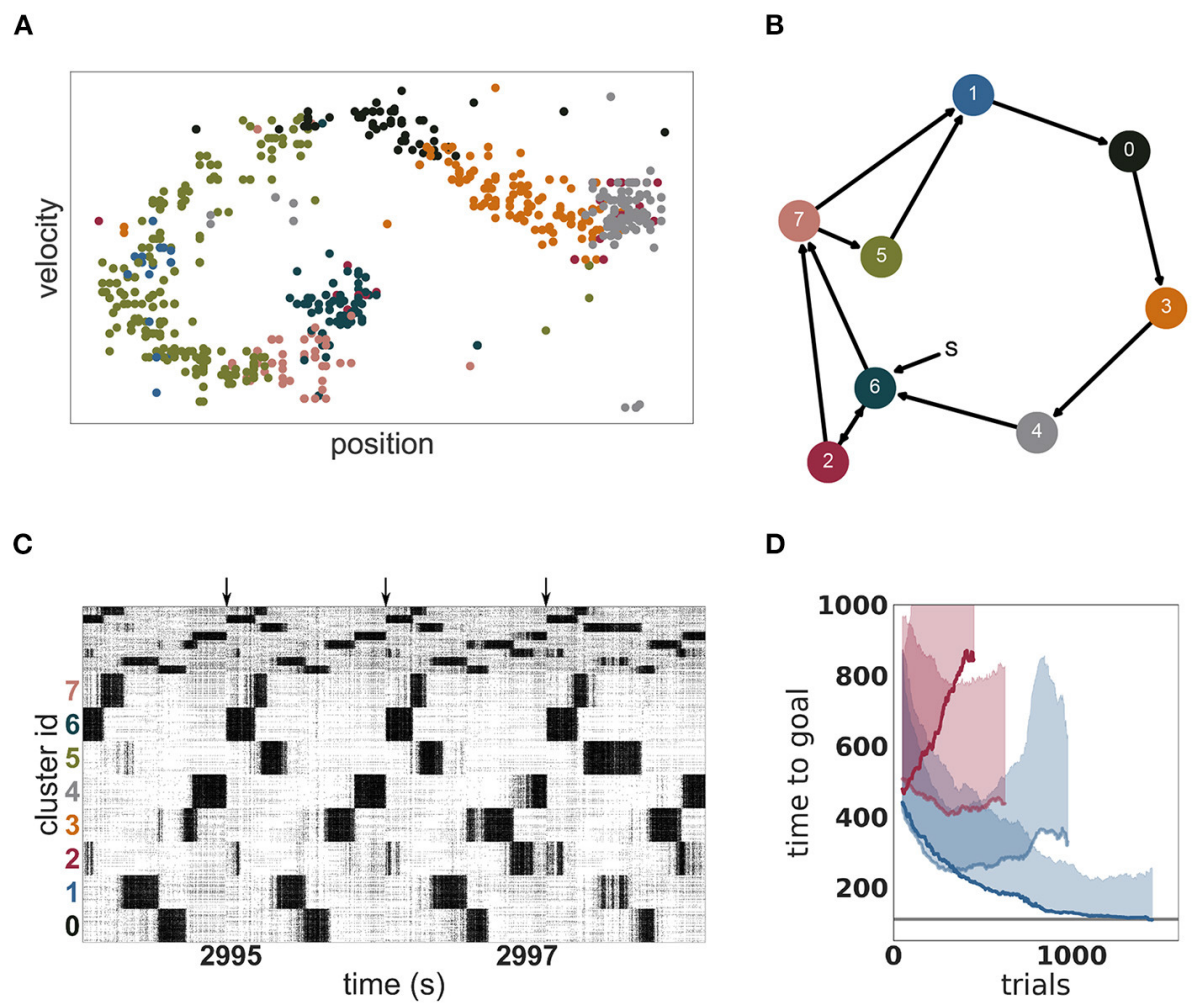
Dynamics and performance of the model during the Mountain Car task. **(A)** Each dot represents the reconstructed position and velocity of the car in the input space during one trial (corresponding to the last trial of **D**). The color of the dots correspond to the most active cluster during this velocity-position pair. **(B)** Most common sequence of active clusters after training. The sequence of each trial usually starts with cluster 6 or 2 and ends on cluster 4. **(C)** Raster plot of the last seconds of the simulation of the Mountain Car task. Arrows indicate the approximate start of a new trial. **(D)** Performance of unclustered (pale) and clustered (dark) networks. Curves give the running median over 10 trials, shaded areas indicate the corresponding standard deviation. Blue and red curves indicate plastic and static input projections, respectively.

An example of such an acquired environmental representation is illustrated in Figure 8A for a single trial after convergence of performance. The mapping of clusters to portions of the two dimensional task space can be clearly seen. Due to this mapping, the clusters are activated in a stereotypical sequence, as is visualized as a directed graph for a specific instantiation in Figure 8B and is also readily apparent in the spiking activity displayed in Figure 8C. For this instantiation, clusters 6 or 2 represent the starting positions and are therefore activated first in the sequence. Cluster 4 represents the goal position and is activated last in the sequence.

The emergent representation of the environment in the representation layer provides a basis for the actor-critic architecture to evaluate states and learn an effective policy, (see Figure 8D). By comparing with Figure 7, it is clear that a mutual bootstrapping of environmental representation and performance is taking place. The better the performance, the more time the agent spend in advantageous dynamical states, causing the representation layer to map these states more effectively to clusters. Complementarily, the sharper the partition of the environment into useful states, the easier it is for the agent to learn appropriate actions, causing an increase in performance.

OpenAI Gym describes the task as solved if the agent needs less than 110 timesteps to find the goal in 100 consecutive episodes. Our model approaches this threshold within 1, 000 episodes (see Figure 8D) but for two reasons we can not claim to have solved the task. First, we adapted the environment in two minor ways as described in section 2.3.3 in order to give our agent more time to explore the environment. Second, although our model frequently finds the goal in less than 110 timesteps, it does not do this consistently. The model is constantly exploring, learning and changing its policy which results in a substantially sub-optimal result in a few trials. Over the last 100 trials, it found the goal in under 110 timesteps on 59 trials, with a median of 108 and an average of 164 timesteps, respectively. As a side remark, humans cannot solve the task easily either, as reaching the goal 100 times in a row and consistently, under 110 timesteps is challenging and tiring.

As with the MNIST task, it is the combination of a clustered network and plastic input synapses that yields the best results. As shown in Figure 8D, an unclustered network with plastic synapses shows initial improvement on the task but saturates early at just over 200 timesteps, and then becomes gradually worse (median 329, average 416 timesteps over the last 100 episodes). An unclustered network with static input synapses does not show appreciable change in performance even after many iterations (median 437, average 541). Unlike the MNIST task, for the Mountain Car task the worst performance is demonstrated by the clustered network with static synapses (median 844, average 1, 086). In this configuration, cluster switching becomes rare, and so the network takes longer to reach the goal.

Also in common with the MNIST task, response variability, as measured by the change in coefficient of variation is the best indicator of performance. For the networks with plastic input synapses, the CV of the clustered network increases during the simulation from 0.43 to 3.99 (whilst the firing rate increases mildly from 15.5 to 18.8 spikes per second); the CV of the unclustered network increases from 0.93 to 2.08 (whilst the firing rate increases substantially from 8.8 to 22.8 spikes per second). For the networks with static input synapses, the CV of the unclustered network stays constant at 0.78 whereas the CV of the clustered network decreases from 0.69 to 0.55.

## 4. Discussion

In this work, we present a spiking neural network model for unsupervised feature extraction and reinforcement learning using clustered spiking networks and Hebbian plasticity with synaptic scaling. We demonstrate that this combination is able to extract complex features and resolve non-linearities in the input elegantly. The XOR task (section 4) demonstrated that the presence of unsupervised plasticity on the input projections can boost the performance of a reservoir computing system, even in a very small and simplified network. Whereas this simple network with static input projections was unable to transform the input into a linearly separable projection required to resolve the task, simply adding a local and entirely unsupervised learning rule, compatible with biological findings, allows it to adequately do so.

Using the full version of the model, with a random balanced network as the representation layer, we showed that the combination of unsupervised learning on the input projections with clustered connectivity boosts the performance of a reinforcement learning approach. On the reduced MNIST task (section 3.2), the clustered network with unsupervised plasticity learns faster and achieves a better performance than an unclustered network. Networks without input plasticity performed at chance level. Examining the structure of the input projections revealed that the clusters had specialized for particular sub-categories of the input space (e.g., *two*'s with and without a loop, see Figure 5). For all cases analyzed, this projection specificity remains stable after learning as the synaptic weights converge to a stationary state.

We conclude that, for this task, the pre-existence of clusters supports the self-organization of representation by permitting feature specialization to develop, i.e., if the input is adequately projected onto clusters of strongly connected neurons, competition between the clusters allows them to become tuned to the relevant input features driving them, such that the system learns task-relevant mappings faster and in a more robust manner. In addition, subtle variations in the input feature space can be discerned, if the representation layer comprises a sufficient number of internal clusters, which become specialized for class membership as well as intra-class variance. These specialized classifiers learned by the clusters thus allow a faster and more robust learning performance. Note that this specialization is an emergent property of the combination of the clusters and the synaptic plasticity between the input and the representation layers, and doesn't stem from explicit class labels, as none were provided.

Overall, based on the results obtained on the XOR and the MNIST tasks, we can differentiate the contributions of the features that give the system its functionality. Unsupervised learning in the input projections appears to sharpen representational specificity, generating linearly separable embeddings of the input data. Complementarily, clustered connectivity in the representation layer results in a reduction of the system's effective dimensionality, thus circumventing the variation introduced due to spiking dynamics. By partitioning the representation layer into several homogeneous clusters, the activation of a cluster can be seen as a coarse approximation of a single firing rate unit, thus reducing the effective dimensionality in the representation layer and operating as a mechanism to reduce variability in the responses of individual neurons. In fact, if the representation layer were composed of uncoupled rate neurons, random input projections would suffice to resolve non-linear separability problems, by generating neurons with mixed selectivity to the input features (see e.g., Rigotti et al., 2010; Fusi et al., 2016). However, our results show that the effects introduced by clustered structure are insufficient; unsupervised learning is required to sharpen the selectivity of single units and clusters, resulting in representations that go beyond a simple linear separation of the input data, to enable specialization for minor variations in the input features, as demonstrated by the MNIST task (see Figure 5).

A spiking implementation of an actor-critic reinforcement learning architecture enables our model to solve tasks with sparse and delayed feedback, exemplified by the time- and space-continuous Mountain Car problem. On this task, the clusters became specialized for particular regions of the two dimensional input space, for which the appropriate action could easily be learned. This specialization develops independently of the task feedback that drives learning (RPE), relying only on the statistical structure of the input data. However, in contrast to MNIST, the stream of input data is influenced by the actions selected by the agent. As the agent learns the task, certain regions of the state space are visited more often, and the order in which they are visited becomes more predictable. Cluster activations after learning reflect the frequency and temporal order with which different regions in the input space are visited, thereby reflecting a task-oriented, rather than comprehensive (grid-like), discretization of the state-space. This self-organized, task-driven discretization is efficient in its use of the available resources in that only task-relevant partitions are learned and no representational dimensions are wasted in covering irrelevant regions of state-space. In contrast, the commonly used *ad hoc* grid-like partitioning would entail that a proportion of neurons were assigned to cover regions of the input space that are not relevant for the task and may never be visited at all.

Thus, the network model simultaneously learns an appropriate partition of the task structure, the value of each partition and the optimal action to take in it, and it does so in a resource-efficient manner. Once learning has converged, the model cycles through a stereotypical sequence of cluster activations in order to solve the task, and achieves a much better performance than the corresponding unclustered network, frequently (but not consistently) finding the goal within the time limit set by OpenAI Gym to consider the task solved.

We emphasize that the motivation for the network exploration in this study is not to achieve optimal performance on the considered tasks, but rather to gain new insights into how the brain might develop adequate representational spaces, under known biophysical constraints and without a priori knowledge of the task format. There certainly exist alternative spiking network models that can outperform ours (for MNIST, see, for example, Habenschuss et al., 2012; Diehl and Cook, 2015), typically by introducing non-biological model assumptions on architecture, dynamics, or learning rules. Similarly, one could consider variants of our model that use fewer neurons, such as by replacing the clusters in the representational layer with efficient WTA-circuits or individual firing rate units that would correspond to the activity within the cluster. However, such variants would detract from the key finding of our study, which is that the computational properties of the balanced random network are enhanced by the introduction of the biologically motivated features of unsupervised plasticity and clustered connectivity. Of greater interest would be variants that take further steps toward biological verisimilitude (for instance, with respect to the generation of the reward prediction error) without notable loss of performance, or those that increase performance at the same level of biological plausibility.

The *representation layer* is left untrained. This has two important ramifications. First, the representation layer does not reflect any previous knowledge or assumptions about an appropriate partitioning of the input space on the part of the modeler, unlike previous approaches with hard-wired place cells or radial basis functions. Second, the network can be re-used to represent different patterns and data sets. As long as the input projections are adapted to the properties of the data, no further modifications are required within our set-up; the exact same network is used for both the MNIST and Mountain Car tasks requiring no modifications of its internal architecture.

The ability to adequately represent data is a critical step for any learning system to be able to detect statistically repeating patterns. Depending on the task and data set considered, it might be important, for example, to extract features that allow a scale- or rotation-invariant representation or to reduce the input dimensionality making it simpler to process. The most commonly known method for dimensionality reduction and feature extraction is principal component analysis (PCA), but many others exist, such as linear discriminant analysis (LDA, Mika et al., 1999), independent component analysis (ICA, Comon, 1994; Hyvärinen and Oja, 2000) or scale-invariant feature transform (SIFT Lowe, 1999). Modern techniques based on convolutional deep networks (CNN, e.g., Mnih et al., 2015) have also proven valuable for feature extraction, particularly in the domain of computer vision, whereby complex, hierarchical feature dependencies are gradually extracted through error back-propagation.

All these methods are powerful tools, but their biological plausibility is limited or non-existent. As such, they are not appropriate models for studying learning in the mammalian brain. A more plausible approach is based on “competitive learning” as described in Hertz et al. (1991), which employs Oja's rule (modified Hebbian learning with multiplicative normalization). This model was shown to perform PCA, constituting a powerful, biologically-plausible alternative for feature extraction and dimensionality reduction (Oja, 1982), see also Qiu et al. (2012) for a detailed review on neural networks implementing PCA or non-linear extensions of PCA. In a recent study, competitive unsupervised learning applied to the lower layers of a feedforward neural network was shown to successfully lead to good generalization performance on the MNIST dataset (Krotov and Hopfield, 2019). However, despite providing further validation to the claim that biological compatibility need not be sacrificed for adequate computational performance, these studies still rely on implausible mechanisms, such as decoupled processing layers (i.e. purely feedforward connectivity) and simplified processing units (sigmoid or rectified linear units).

Alternative, related instantiations have been proposed in the literature. Employing Hebbian learning with synaptic scaling to the internal, recurrent connections was shown to be mathematically equivalent to non-negative matrix factorization (Carlson et al., 2013) and to allow for the development and maintenance of multiple internal memories, without catastrophic interference effects (Auth et al., 2020). On the other hand, applying similar learning rules exclusively to the input projections (as we have), leads to systems that perform operations similar to ICA (Jonke et al., 2017). These examples employ a conceptually similar learning rule in small network architectures (8, 500, and 900 neurons, respectively). In such small networks, single neurons can become highly tuned and influential enough to reliably implement the competition needed by inhibiting the rest of the network. For larger networks, however, a coordinated activation of relatively large sub-populations is necessary to achieve this internal competition. The combination of Hebbian learning with lateral inhibition, as introduced by Diehl and Cook (2015) and Querlioz et al. (2013), can comply with this requirement, leading to highly accurate representations, as demonstrated by the remarkably high accuracy achieved by this model on the MNIST dataset. Competition is, in that case, instantiated by inhibitory projections, which actively silence the unstimulated excitatory neurons. Homeostasis, in the form of a neuron-intrinsic property (adaptive threshold), then ensures that all neurons have a fair chance to compete. Our model, on the other hand, achieves this competition effect as a result of the clustered connectivity (see section 3.2), without sacrificing the biological compatibility of the network's architecture (namely sparse recurrent interactions and distributed, population-level representations) and thus highlights the functional relevance of structurally constrained recurrent connections.

The existence of the clustered synaptic connectivity investigated in our study is an important feature of cortical circuits (Song et al., 2005; Perin et al., 2011), reflecting either evolutionary constraints or life-long learning and synaptic plasticity (Litwin-Kumar and Doiron, 2014; Zenke et al., 2015). It has been shown that it can account for pervasive phenomena in cortical microcircuits, such as a modulation of the timescales of intrinsic firing fluctuations and their variability (Litwin-Kumar and Doiron, 2012), a drop in effective dimensionality of population responses during active processing, as well as the emergence and modulation of stimulus-specific metastable states and structured transitions among them (Mazzucato et al., 2016).

Cortical microcircuits are known to rapidly switch among different active states, characterized by markedly different dynamical properties and critically modulating stimulus processing and the fidelity of stimulus representations (Duarte, 2015; Gutnisky et al., 2017). While the majority of the studies on the matter focus on the relation between ongoing and evoked activity and/or trial-to-trial variability (see, e.g., Churchland et al., 2010, and references therein), much less is known about learning-induced changes in circuit responsiveness and cortical states (Kwon, 2018). A common observation is that the onset of a stimulus reduces the variability of the elicited responses (Churchland et al., 2010) by coalescing population activity onto a low-dimensional manifold (Jazayeri and Afraz, 2017; Remington et al., 2018). One would thus expect that during learning, these representational structures become sharper and this specialization would be reflected in a reduction in response variability.

Our results demonstrate an increase in spike-train variability across the population, but, in this situation, this is clearly a result of modular specialization. Tuned sub-populations of neurons (within a cluster) fire strongly for short periods of time and sparsely when the cluster they belong to is inactive. This skews the distributions of inter-spike intervals, greatly increasing its variance and reducing its mean, resulting in a larger CV. However, trial-to-trial variability is clearly decreased after learning (as can be seen in Figure 4B). The observed increase in population-level variability in this set-up thus reflects a more constrained dynamical space and is a consequence of switching between highly active, specialized clusters. Thus, based on these observations from our model, we can expect that learning-induced modulations of population dynamics would result in an increase in population-level variability (as measured, for example by the CV). Having experimental access to a large enough population of responsive neurons would allow us to observe the formation of an increasingly restrictive dynamical space, whereby task-relevant variations would be imprinted in firing co-variation among strongly connected neuronal clusters (in line with Jazayeri and Afraz, 2017).

Our model constitutes an important step forward in the domain of unsupervised feature extraction, potentially leading to flexible learning algorithms, which can be used on large computational domains without requiring task-specific adjustments to the structure of the system. Because of its biological inspiration and plausibility, both in the learning rules and internal architecture, the model allows us to make predictions about the dynamic properties of internal representations and thus has the potential to lead to a better understanding of the principles underlying learning in the mammalian brain.

Capitalizing on these features, further work is required to clarify the extent of the model's functional and biological relevance. For example, in this study we showed that the presence of clusters improved the performance of the network, but the cluster size relative to network size and cluster membership were arbitrarily assigned. We have demonstrated that the number of internal clusters that specialize the representation layer are related to task constraints (see Figure 6) and should be sufficient to capture intrinsic variation in the input data and enforce the necessary competition. However, beyond an optimum value, adding more clusters leads to redundant representations and decreases overall performance. It is thus reasonable to expect that the optimal parameter configuration depends on the demands of the task. Future work should thus establish systematic relations between the number and size of internal network clusters, their functional impact relative to task demands as well as their effects on the observed dynamics. Furthermore, as we could use the same representational layer for different tasks, this suggests that this type of architecture may be suitable for multi-task learning. It remains to be seen how quickly the network can be re-trained on a different task, and whether such a re-mapping induces catastrophic forgetting of the first task, or permits a palimpsest of functionally relevant cluster mappings to be established.

## Data Availability Statement

All simulation scripts and datasets analyzed in this study are publicly available and can be found here: https://fz-juelich.sciebo.de/s/iSAZ7be2prtCCXS.

## Author Contributions

This study was conceived and designed by PW, RD, and AM. All models, software and simulations were implemented by PW. Data analyses and figures were performed by PW and RD. The manuscript was written jointly by all authors.

## Conflict of Interest

The authors declare that the research was conducted in the absence of any commercial or financial relationships that could be construed as a potential conflict of interest.

## Footnotes

^1^http://yann.lecun.com/exdb/mnist/

^2^https://github.com/openai/gym/wiki/MountainCar-v0

^3^https://fz-juelich.sciebo.de/s/iSAZ7be2prtCCXS
