# Supplementary material for: Unsupervised Learning and Clustered Connectivity Enhance Reinforcement Learning in Spiking Neural Networks
Source: Front Comput Neurosci. 2021 Mar 4;15:543872. doi: 10.3389/fncom.2021.543872 (PMC7970044; doi:10.3389/fncom.2021.543872)
Supplement: Supplementary file 1 [file Data_Sheet_1.PDF]

Supplementary Material

1 Network description

A	Model summary
**Populations**	Input, representation (excitatory and inhibitory) and output
**Connectivity**	Input to representation layer, Random recurrent connections in representation layer (with or without clusters), representation to output connections
**Neuron model**	Leaky integrate-and-fire, fixed voltage threshold, fixed absolute refractory time, exponential current-based synapses
**Synapse model**	Input: Hebbian with quadratic normalization term (Tetzlaff et al., 2013)<br>Output: Hebbian with quadratic normalization and neuromodulatory third factor<br>Reservoir: static
**Input**	Independent stochastic background input, rate-coded input signal (independent Poisson processes)
**Measurements**	Spiking activity, firing rates

B	Populations	
**Name**	**Elements**	**Size**
Background input	Independent Poisson processes	n_{b} (rate F_{b})
Input	Inhomogeneous Poisson processes	n_{inp} (rate F_{inp})
Reservoir exc	Integrate-and-fire neuron	n_{e}
Reservoir inh	Integrate-and-fire neuron	n_{i}
Output	Integrate-and-fire neuron	n_{out}

C	Connectivity		
**Name**	**Source**	**Target**	**Pattern**
Input Projections	Input	Reservoir	all-to-all, initial weight w_{inp}
EE	Reservoir exc	Reservoir exc	Random pairwise Bernoulli, p = p_{ee}, weight w_{ee}
EI	Reservoir exc	Reservoir inh	Random pairwise Bernoulli, p = p_{ei}, weight w_{ei}
IE	Reservoir inh	Reservoir exc	Random pairwise Bernoulli, p = p_{ie}, weight w_{ie}
II	Reservoir inh	Reservoir inh	Random pairwise Bernoulli, p = p_{ii}, weight w_{ii}
Output Projections	Reservoir exc	Output	all-to-all, initial weight w^{μ}_{out} $w^{\sigma}_{\mathrm{out}}$
Background	Background input	Output	all-to-all, weight w_{b}
Output	Output	Output	all-to-all, weight w_{wta}, delay d_{wta}

D	Neuron model
**Name**	`iaf_psc_exp`
**Subthreshold dynamics**	if $(t > t^* + \tau_{\mathrm{ref}})$ $\dot{V_m} = -V_m/\tau_m + I/C$ else $V_m(t) = V_{\mathrm{reset}}$<br>Synaptic inputs evoke exponential post-synaptic currents with time constant τ_{s}
**Spiking**	If $V(t-) < V_{\mathrm{th}}$ OR $V(t+) \geq V_{\mathrm{th}}$<br>1. set $t^* = t$<br>2. emit spike with time stamp t^*

E	Plasticity	
**Source**	**Target**	**Equation**
Input	Reservoir	$\Delta w_{ji} = \mu \left(F_i F_j + \kappa (F^{\mathrm{T}} - F_j) w_{ji}^2 \right)$
Reservoir	Output	$\Delta w_{ji} = \mu \left((D - b_{\mathrm{D}}) F_i F_j + \kappa (F^{\mathrm{T}} - F_j) w_{ji}^2 \right)$

F	Input	
**Source**	**Target**	**Description**
Poisson Generator	Output	Independent background noise, rate F_{b}, weight w_{b}
Inhomogeneous Poisson	Reservoir	Time-dependent rate (task-specific), initial weight w_{inp}

G	Measurements
Spiking activity (input, reservoir and output)	

Table 1: Tabular description of network model after Nordlie et al., 2009.

1.1 Clustered balanced random network

A		Populations
**Name**	**Value**	**Description**
n_{inp}	task-dependent	Number of input neurons
n_{e}	4000	Number of excitatory neurons in representation layer
n_{i}	1000	Number of inhibitory neurons in representation layer
n_{out}	3	Number of output neurons
n_{b}	1	Number of background sources
**B**		**Connectivity**
**Name**	**Value**	**Description**
w_{inp}	learned (initial value)	Amplitude of excitatory input projections
$p_{\text{ee}}^{\text{intra}}$	0.2	Connection probability for intra-cluster excitatory to excitatory connections
$w_{\text{ee}}^{\text{intra}}$	2.28 pA	Amplitude of intra-cluster excitatory to excitatory connections
$p_{\text{ee}}^{\text{extra}}$	0.2	Connection probability for extra-cluster excitatory to excitatory connections
$w_{\text{ee}}^{\text{extra}}$	0.19 pA	Amplitude of extra-cluster excitatory to excitatory connections
$p_{\text{ei}}^{\text{intra}}$	0.5	Connection probability for intra-cluster inhibitory to excitatory connections
$w_{\text{ei}}^{\text{intra}}$	1.33 pA	Amplitude of intra-cluster inhibitory to excitatory connections
$p_{\text{ei}}^{\text{extra}}$	0.5	Connection probability for extra-cluster inhibitory to excitatory connections
$w_{\text{ei}}^{\text{extra}}$	0.19 pA	Amplitude of extra-cluster inhibitory to excitatory connections
$p_{\text{ie}}^{\text{intra}}$	0.5	Connection probability for intra-cluster excitatory to inhibitory connections
$w_{\text{ie}}^{\text{intra}}$	−6.35 pA	Amplitude of intra-cluster excitatory to inhibitory connections
$p_{\text{ie}}^{\text{extra}}$	0.5	Connection probability for extra-cluster excitatory to inhibitory connections
$w_{\text{ie}}^{\text{extra}}$	0.91 pA	Amplitude of extra-cluster excitatory to inhibitory connections
$p_{\text{ii}}^{\text{intra}}$	0.5	Connection probability for intra-cluster inhibitory to inhibitory connections
$w_{\text{ii}}^{\text{intra}}$	−9.24 pA	Amplitude of intra-cluster inhibitory to inhibitory connections
$p_{\text{ii}}^{\text{extra}}$	0.5	Connection probability for extra-cluster inhibitory to inhibitory connections
$w_{\text{ii}}^{\text{extra}}$	1.32 pA	Amplitude of extra-cluster inhibitory to inhibitory connections
w_{out}^{μ}	5.0 pA	Mean reservoir to output connection amplitude
w_{out}^{σ}	1.44	Standard deviation of reservoir to output connection amplitudes
w_{wta}	−3000 pA	Amplitude of output connections
d_{wta}	$[1, 3]$ ms	Delay of output connections
**D**		**Representation layer neurons**
C	1 pF	Membrane capacitance
V_{th}	20 mV	Fixed firing threshold
τ_{m} (exc)	20 ms	Membrane time constant (exc neurons)
τ_{m} (inh)	10 ms	Membrane time constant (inh neurons)
τ_{s}	2 ms	Synaptic time constant
I_{e} (exc)	0.825 pA	Bias current (exc neurons)
I_{e} (inh)	0.745 pA	Bias current (exc neurons)
t_{ref}	5 ms	Absolute refractory period
**E**		**Output layer neurons**
C	250 pF	Membrane capacitance
V_{th}	15 mV	Fixed firing threshold
τ_{m}	20 ms	Membrane time constant
τ_{s}	2 ms	Synaptic time constant
I_{e}	0 pA	Bias current
t_{ref}	2 ms	Absolute refractory period

Table 2: Model parameters for clustered networks.

1.2 Unclustered balanced random network

A	Populations	
**Name**	**Value**	**Description**
n_{inp}	task-dependent	Number of input neurons
n_{e}	4000	Number of excitatory neurons in representation layer
n_{i}	1000	Number of inhibitory neurons in representation layer
n_{out}	3	Number of output neurons
**B**	**Connectivity**	
**Name**	**Value**	**Description**
w_{inp}	task-dependent	Amplitude of excitatory input projections
p_{ee}	0.2	Connection probability for excitatory to excitatory connections
w_{ee}	0.45 pA	Amplitude of excitatory to excitatory connections
p_{ei}	0.5	Connection probability for inhibitory to excitatory connections
w_{ei}	0.33 pA	Amplitude of inhibitory to excitatory connections
p_{ie}	0.5	Connection probability for excitatory to inhibitory connections
w_{ie}	-1.59 pA	Amplitude of excitatory to inhibitory connections
p_{ii}	0.5	Connection probability for inhibitory to inhibitory connections
w_{ii}	-2.31 pA	Amplitude of inhibitory to inhibitory connections
w^{μ}_{out}	5.0 pA	Mean reservoir to output connection amplitude
$w^{\sigma}_{\mathrm{out}}$	1.44	Standard deviation of reservoir to output connection amplitudes
w_{wta}	-3000 pA	Amplitude of output connections
d_{wta}	$[1, 3]$ ms	Delay of output connections
**D**	**Representation layer neurons**	
C	1 pF	Membrane capacitance
V_{th}	20 mV	Fixed firing threshold
τ_{m} (exc)	20 ms	Membrane time constant (exc neurons)
τ_{m} (inh)	10 ms	Membrane time constant (inh neurons)
τ_{s}	2 ms	Synaptic time constant
I_{e} (exc)	0.825 pA	Bias current (exc neurons)
I_{e} (inh)	0.745 pA	Bias current (exc neurons)
t_{ref}	5 ms	Absolute refractory period
**E**	**Output layer neurons**	
C	250 pF	Membrane capacitance
V_{th}	15 mV	Fixed firing threshold
τ_{m}	20 ms	Membrane time constant
τ_{s}	2 ms	Synaptic time constant
I_{e}	0 pA	Bias current
t_{ref}	2 ms	Absolute refractory period

Table 3: Model parameters for unclustered networks.

2 Task description

XOR		
**Name**	**Value**	**Description**
F_{inp}	$[10, 1000]$ Hz	Range of input firing rates
w_{inp}	50 ± 33 pA	Initial amplitude of input projections
**Input Plasticity**		
μ	0.0001	Global learning rate for input synapses
κ	0.15	Ratio of synaptic scaling to Hebbian learning
F^{T}	1.0	Homeostatic set point (target firing rate)

MNIST (8 cluster)		
**Name**	**Value**	**Description**
F_{b}	95 kHz	Background firing rate
w_{b}	2 pA	Amplitude of background input synapses
**Input**		
F_{inp}	$[0, 20]$ Hz	Range of input firing rates
w_{inp}	3 pA	Initial amplitude of input projections
**Input Plasticity**		
μ	0.0001	Global learning rate
κ	0.19	Ratio of synaptic scaling to Hebbian learning
F^{T}	0.4	Homeostatic set point (target firing rate)
**Output Plasticity**		
μ	0.000001	Global learning rate
κ	0.0	Ratio of synaptic scaling to Hebbian learning
F^{T}	0.0	Homeostatic set point (target firing rate)
b_{D}	9.7	Baseline dopaminergic concentration

Mountain Car (8 cluster)		
**Name**	**Value**	**Description**
F_{b}	95 kHz	Background firing rate
w_{b}	2 pA	Amplitude of background input synapses
**Input**		
F_{inp}	$[0, 35]$ Hz	Range of input firing rates
w_{inp}	2 pA	Initial amplitude of input projections
**Input Plasticity**		
μ	0.0001	Global learning rate
κ	0.12	Ratio of synaptic scaling to Hebbian learning
F^{T}	0.1	Homeostatic set point (target firing rate)
**Output Plasticity (actor)**		
μ	0.00001	Global learning rate
κ	0.0	Ratio of synaptic scaling to Hebbian learning
F^{T}	0.0	Homeostatic set point (target firing rate)
b_{D}	9.7	Baseline dopaminergic concentration
**Output Plasticity (critic)**		
μ	0.00001	Global learning rate
κ	0.0	Ratio of synaptic scaling to Hebbian learning
F^{T}	0.0	Homeostatic set point (target firing rate)
b_{D}	9.7	Baseline dopaminergic concentration
